# Iowa Medical Journal

**Published:** 1853-09

**Authors:** 


*Iowa Medical Journal*, conducted by the Faculty of the Medical Department of the Iowa University, Keokuk, Iowa. Published monthly at $2 per annum in advance.

We have received no news of the death of our quondam neighbor, the Medico Chirurgical Journal; we suppose, therefore, it still lives. We hope the University will make their new organ a better and more substantial Journal than its predecessor. Their appearance is creditable and the matter good. We cordially wish them success. J.

